# Role of Gap Junction Protein Connexin43 in Astrogliosis Induced by Brain Injury

**DOI:** 10.1371/journal.pone.0047311

**Published:** 2012-10-23

**Authors:** Nicolas Theodoric, John F. Bechberger, Christian C. Naus, Wun-Chey Sin

**Affiliations:** Department of Cellular and Physiological Sciences, Life Sciences Institute, The University of British Columbia, Vancouver, British Columbia, Canada; Virginia Commonwealth University, United States of America

## Abstract

Astrogliosis is a process that involves morphological and biochemical changes associated with astrocyte activation in response to cell damage in the brain. The upregulation of intermediate filament proteins including glial fibrillary acidic protein (GFAP), nestin and vimentin are often used as indicators for astrogliosis. Although connexin43 (Cx43), a channel protein widely expressed in adult astrocytes, exhibits enhanced immunoreactivity in the peri-lesion region, its role in astrogliosis is still unclear. Here, we correlated the temporal and spatial expression of Cx43 to the activation of astrocytes and microglia in response to an acute needle stab wound *in vivo*. We found large numbers of microglia devoid of Cx43 in the needle wound at 3 days post injury (dpi) while reactive astrocytes expressing Cx43 were present in the peripheral zone surrounding the injury site. A redistribution of Cx43 to the needle site, corresponding to the increased presence of GFAP-positive reactive astrocytes in the region, was only apparent from 6 dpi and sustained until at least 15 dpi. Interestingly, the extent of microglial activation and subsequent astrogliosis in the brain of Cx43 knockout mice was significantly larger than those of wild type, suggesting that Cx43 expression limits the degree of microgliosis. Although Cx43 is not essential for astrogliosis and microglial activation induced by a needle injury, our results demonstrate that Cx43 is a useful marker for injury induced astrogliosis due to its enhanced expression specifically within a small region of the lesion for an extended period. As a channel protein, Cx43 is a potential *in vivo* diagnostic tool of asymptomatic brain injury.

## Introduction

The host response to a disease state in the brain such as injury or cancer often involves a coordination of multiple cell types including astrocytes, microglia and oligodendrocyte precursor cells (OPCs), also known as NG2-glia [1], [2], [3], [4], [5]. In particular, microglia which are the inflammatory resident cells of the brain undergo a morphological change from a stellate to an amoeboid phenotype [6], [7]. Astrocytes similarly undergo hypertrophic morphological changes coupled with enhanced glial fibrillary acidic protein (GFAP) expression in a process known as reactive gliosis [8], [9], [10], [11]; they are also the main component of the glial scar that encase the brain lesion, thereby isolating the injury sites from the surrounding brain tissues [9]. Indeed increased immunoreactivity of GFAP, and to a lesser extent vimentin and nestin, has been widely used as definitive markers for astrogliosis [9]. Nevertheless, many more signaling molecules have been shown to be upregulated in reactive astrocytes [11].

The gap junction protein connexin43 (Cx43), widely expressed in adult astrocytes [12], [13], has been detected in regions with astrogliosis induced by various brain pathologies including brain ischemia and epilepsy [14], [15], [16], [17], [18], [19]. As gap junctions form channels that allow passage of small molecules such as ATP and glutamate between adjacent cells [20], they are especially suited to play a pivotal role in intercellular communication in a diseased state [21], [22]. In addition, gap junction proteins can also form hemichannels that connect the cytoplasm directly to the extracellular space [23]. In this regard, the ATP release by Cx43 has been proposed to have a major role in the inflammatory response of the brain [24]. To clarify the role of Cx43 in host inflammatory responses *in vivo*, we used a simple needle wound in the brain to generate a localized injury. We observed the dynamic profile of Cx43 due to the injury mirrored the spatiotemporal distribution of GFAP-expressing astrocytes, as opposed to that of microglia, NG2 or nestin-positive cells. We further showed that absence of Cx43 in the brain promotes astrogliosis, indicating that Cx43 is not required for the formation of reactive astrocytes. Nevertheless, the specific localized enhancement of Cx43 immunoreactivity in a small lesion raised the possibility of using the protein as a marker for detecting early stages of brain pathologies.

## Results

### Activation of Astrocytes and Microglia in Response to a Needle Stab Wound

**Figure 1:**
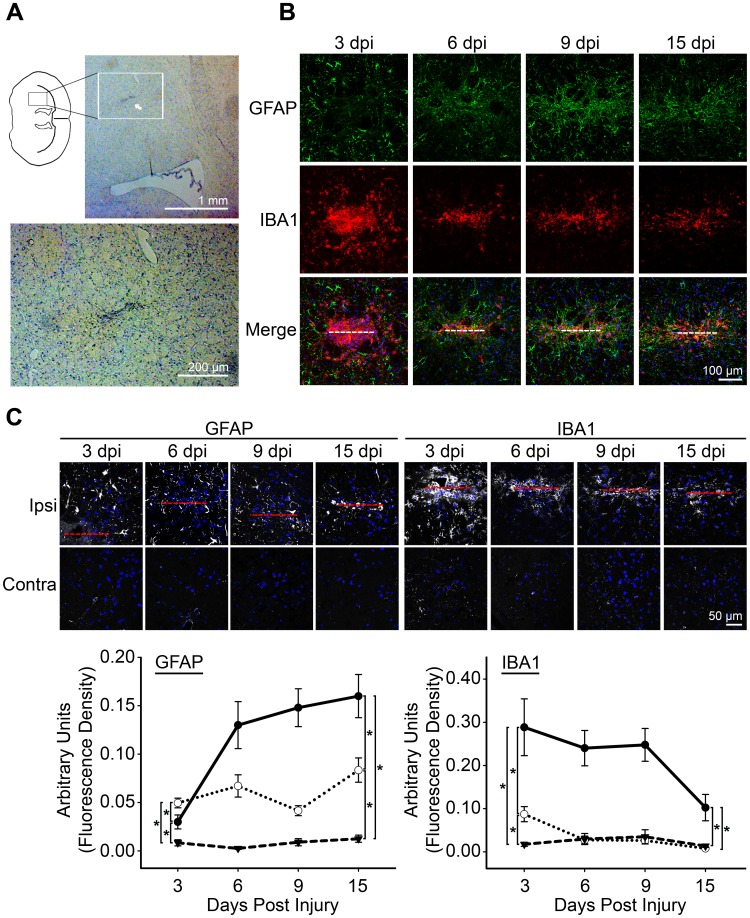
Recruitment of reactive astrocytes and microglia to the needle stab wound. A ) Cresyl violet staining of brain coronal sections showing the needle injury site, as identified by the aggregation of blue nuclei (white arrow). The bottom panel is a magnification of the white box in the upper panel. **B**) A low magnification overview showing the temporal and spatial distribution of GFAP- expressing reactive astrocytes and IBA1-expressing microglia in response to a needle stab lesion in adult mice at 3, 6, 9, and 15 day post injury (dpi). White dotted line indicates location of needle track. **C**) High magnification florescence images of mouse coronal sections at 3, 6, 9, and 15 dpi after needle stab wound showing the distribution of GFAP-positive astrocytes and IBA1-positive microglia in the needle wound (red dotted line) compared to the corresponding position at the contralateral hemisphere. Anti-GFAP and anti-IBA1 were used at a concentration to detect reactive astrocytes or microglia with enhanced GFAP [29] or IBA1 [52] expression, respectively, as demonstrated by the lack of immunoreactivity in the contralateral region. Blue, DAPI nuclei staining. Semi-quantitative analysis of GFAP and IBA1 immunoreactivity was analyzed using Image J (see Materials and Methods) within the needle wound (solid black line), the peripheral zone (dotted line) and the corresponding contralateral region (dashed line). Data were pooled from 4 (3, 6, 9 dpi) and 3 (15 dpi) animals. * = p<0.05.

We used a narrow 33 gauge needle to inflict a simple mechanical injury in the striata of adult wild type mice and monitored the expression of resident glial responses from the host brain (Figure 1A). We first used anti-GFAP at a concentration to visualize only reactive astrocytes [15] expressing an enhanced level of GFAP, and anti-IBA1 [15] to identify microglia, at 3, 6, 9, and 15 days post injury (dpi) (Figure 1B). The accumulation of microglia at the lesion site was obvious at 3 dpi (Figure 1B), indicating rapid microglial activation in response to a needle stab wound. We then measured fluorescence intensity in three distinct regions: a central region that includes the needle lesion, a peripheral region adjacent to the central region and the corresponding contralateral region. The initial accumulation of microglia in the central region at 3 dpi (0.289±0.066) started to decline at 15 dpi (0.103±0.031) but it was still significantly higher than the contralateral region (0.013±0.003) (Figures 1B and 1C). Although a similar significant increase in GFAP staining was observed as early as 3 dpi in the central (0.029±0.007) and the peripheral region (0.050±0.005) when compared to the contralateral region (0.008±0.003) (Figure 1C), GFAP immunoreactivity remained significantly elevated in the peripheral region (0.084±0.01) at 15 dpi throughout the 2-week period (Figure 1C). However, the most dramatic increase of GFAP staining occurred in the central region beginning at 6 dpi (0.130±0.024) which plateaued at 15 dpi (0.160±0.022) in an area indicating possible formation of a glial scar (Figure 1C).

**Figure 2:**
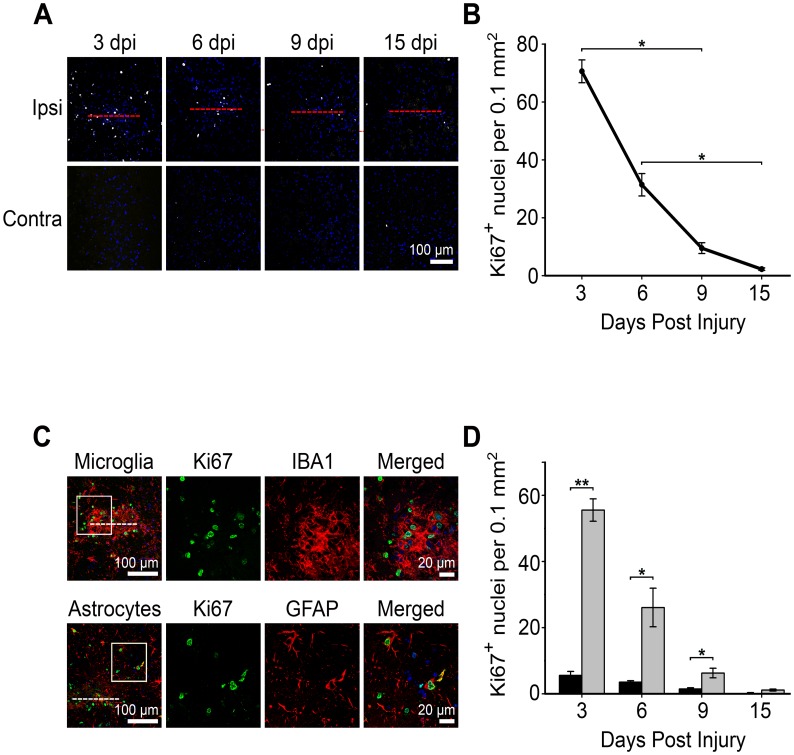
Temporal and spatial distribution of proliferating cells at the lesion site. **A**) Proliferating cells were the most abundant at 3 day post injury (dpi), as revealed by staining the nuclei with anti-Ki67 antibody. Red line denotes position of needle track. **B**) Levels of Ki67-positive nuclei at 3, 6, 9, and 15 dpi. Statistical comparisons were determined by one-way ANOVA on ranks followed by Dunn’s method for pairwise comparison. * = p<0.05. **C**) A large proportion of Ki67-positive cells directly at the needle track stained positive for IBA1, a marker for microglia while a small number of Ki67-positive cells at the needle track periphery were GFAP (a marker for astrocytes) positive. Second to fourth columns show magnified images of the white boxes in the first column. White dotted line indicates location of needle track. Blue, DAPI nuclei staining. **D**) Number of Ki67-positive nuclei co-stained with either astrocytes (GFAP-black bars) or microglia (IBA1-gray bars). ** = P<0.001, *p = <0.05.

Severe gliosis has been associated with the proliferation of astrocytes [9], [25]. In addition, microglia are also known to proliferate in response to CNS injury [1], [26]. We observed the highest number of nuclei staining positive for the cell proliferation marker Ki67 at 3 dpi (Figure 2A and 2B). Most of the Ki67-positive nuclei were located in close vicinity of the needle wound occupied by microglia at 3 dpi (Figure 2C). In addition, we also observed Ki67-positive nuclei in GFAP-positive astrocytes but at a much lower level (Figure 2C and 2D). About 78% of Ki67-positive nuclei were associated with microglia whereas 8% were with astrocytes at 3 dpi (Figure 2D). Taken together, our results show that a simple needle injury, similar to an injection or needle biopsy, was sufficient to induce activation and proliferation of microglia and astrocytes.

### Cx43 Expression at the Needle Stab Wound

**Figure 3:**
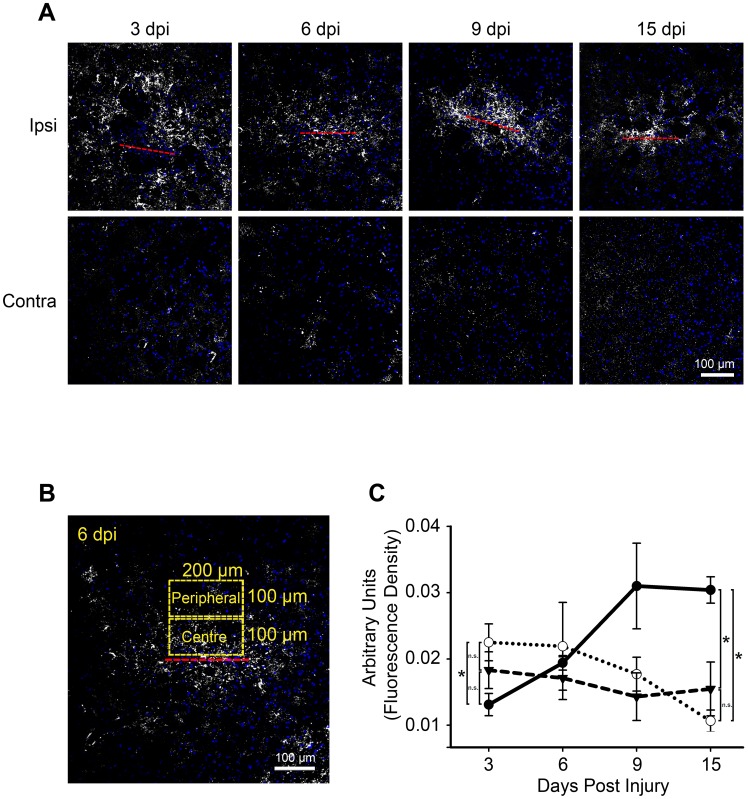
Spatial and temporal distribution of Cx43 protein in response to a needle stab wound injury. **A**) Wild type mouse coronal sections were stained with anti-Cx43 antibodies at 3, 6, 9, and 15 day post injury (dpi). Increased punctate staining typical of Cx43 gap junction plaques [27], [29] directly at the injury site was observed from 6 to 15 dpi, but not at 3 dpi. Some areas of low fluorescence signals in the wound periphery correspond to the myelinated fibers of Pencils of Wilson. Red line denotes position of needle track. Blue, DAPI nuclei staining. **B**) An enlarged image of Cx43 staining at 6 dpi from A) with outlines corresponding to the central and peripheral region of the wound lesion. **C**) The corresponding semi-quantitative analysis of Cx43 immunoreactivity was analyzed using Image J (see Materials and Methods) within the central zone including the needle wound (solid black line), the peripheral zone (dotted line) and the contralateral side (dashed line). Data were pooled from 4 (3, 6, 9 dpi) and 3 (15 dpi) animals. * = p<0.05, n.s. = not significant (p>0.05).

We next determined the spatiotemporal pattern of Cx43 following needle wound injury at 3, 6, 9, and 15 dpi by immunohistochemistry (Figure 3A) and subsequent measurement of the immunofluorescence intensity. Interestingly, a significant reduction of Cx43 expression was observed in the central region directly at the needle track (0.013±0.002) when compared to Cx43 level in the peripheral region (0.023±0.003) at 3 dpi (Figures 3B and 3C). This pattern has also been observed in brain ischemia and spinal cord injuries [27], [28], which may be due to cell death in the immediate vicinity of the lesion. Cx43 immunoreactivity at the central region including the needle track gradually increased from 6 dpi (0.019±0.001) and plateaued at 15 dpi (0.030±0.002) (Figure 3C), which was similar to the spatial and temporal pattern of GFAP following injury (Figure 1). In contrast to GFAP which remained elevated at the periphery, Cx43 immunoreactivity in the peripheral region (0.018±0.003) was reduced to a level equivalent to the contralateral region (0.014±0.004) by 9 dpi (Figures 3A and 3C). Therefore, our observation indicates that Cx43 may be used as a marker to accurately identify lesion sites.

### Reactive Astrocytes within the Needle Lesion Contain Large Cx43 Plaques

**Figure 4:**
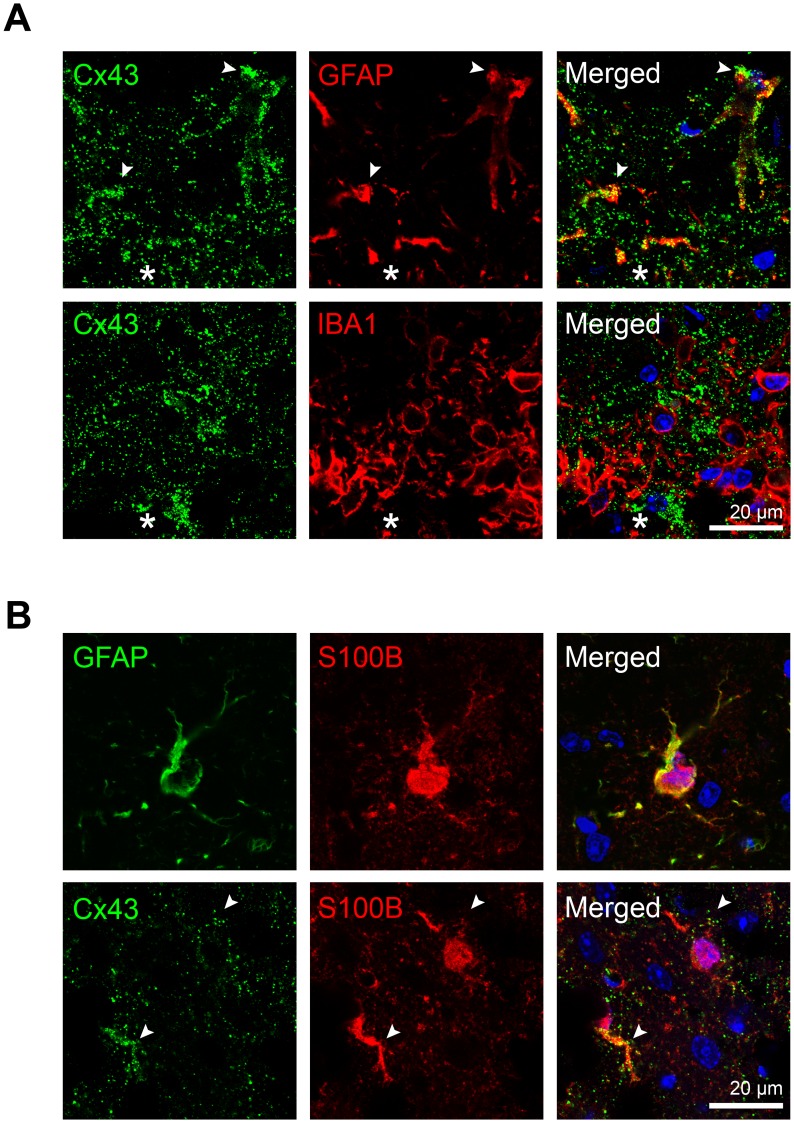
Localization of large Cx43-containing plaques to astrocytes and not to microglia. A ) An increase in localization of Cx43 plaques to GFAP immunoreactivity (white arrowheads) at 6 day post injury (dpi) was observed in wild type mice. Cx43 staining was undetectable in microglia at 6 dpi. **B**) Co-staining of Cx43 with S100β, a cytoplasmic astrocytic protein that colocalized with GFAP confirmed colocalization of Cx43 puncta to S100β-labelled fine processes (arrowheads) in astrocytes. Blue, DAPI nuclei staining.

Our findings suggested that Cx43 mirrored the spatial and temporal kinetics of GFAP-expressing astrocytes. Accordingly, we observed colocalization of Cx43 immunoreactivity to GFAP-positive cells at 6 dpi (Figure 4A) in wild type mice, which is in agreement with previous findings [29], [30], [31]. We confirmed the expression of Cx43 in GFAP-positive astrocytes with an antibody against S100β, a cytoplasmic protein present in astrocytic processes (Figure 4B). In contrast, there was little overlap in staining between Cx43 and IBA1-positive microglia at 6 dpi (Figures 4A), which is consistent with an earlier observation made in a spinal cord injury model [28]. Our data show that the appearance of large Cx43 gap junction plaques during astrogliosis is attributed to the reactive astrocytes in needle stab region.

### Increased Astrogliosis in the Absence of Astrocytic Cx43

**Figure 5:**
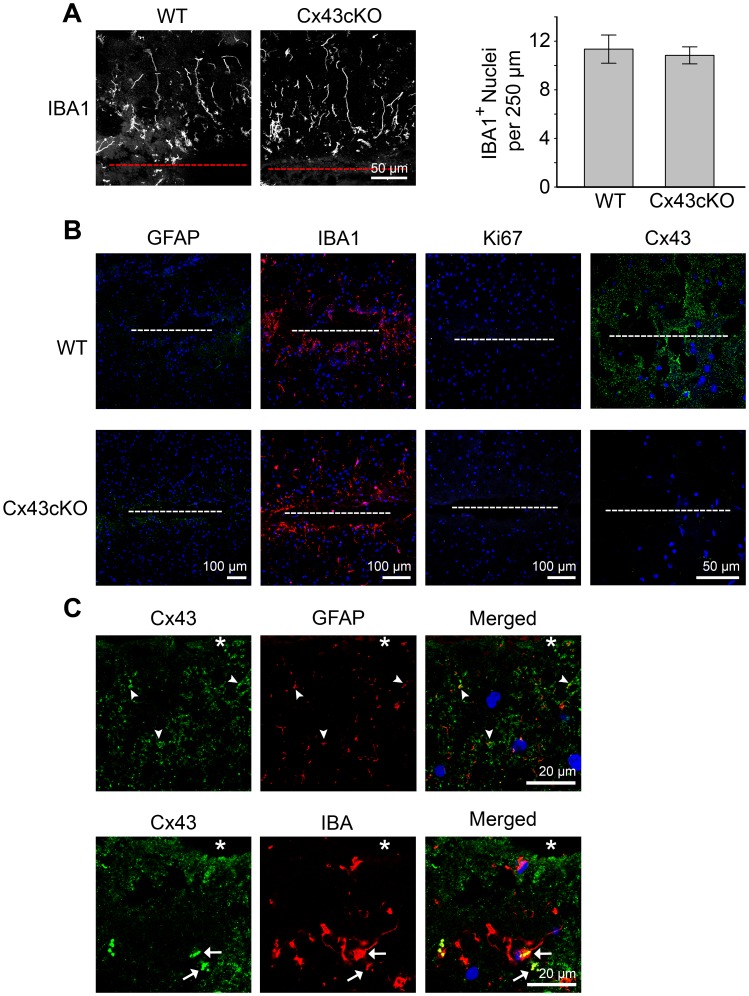
Absence of astrocytic Cx43 does not affect number of microglia at the lesion site. **A**) (*left panel)* A high magnification image showing the extension of processes perpendicular to the wound by IBA1-positive microglia in wild type (WT) and Cx43 deleted (Cx43cKO) brain of GFAP-Cre, Cx43 fl/fl mice at 3 hour post injury (hpi). *(right panel)* Graphical representation showing no difference in the number of DAPI-positive nuclei in the lesion site that co-stained with IBA1 marker in WT and Cx43cKO brain at 3 hpi. **B**) Lack of GFAP-expressing reactive astrocytes and Ki67-positive proliferating cells in response to a needle stab lesion in at 3 hpi in Cx43-expressing WT and Cx43-deficient Cx43cKO brains. Anti-GFAP antibody was used at a concentration to detect only reactive astrocytes with enhanced GFAP expression. **C**) Co-staining of Cx43 with GFAP showed limited localization of Cx43 puncta to GFAP-positive astrocytes (white arrowheads) at 3 hpi surrounding the needle hole (*) in WT mice. Cytoplasmic Cx43 was observed in some IBA1-expressing microglia (white arrows) at 3 hpi. Blue, DAPI nuclei staining.

**Figure 6:**
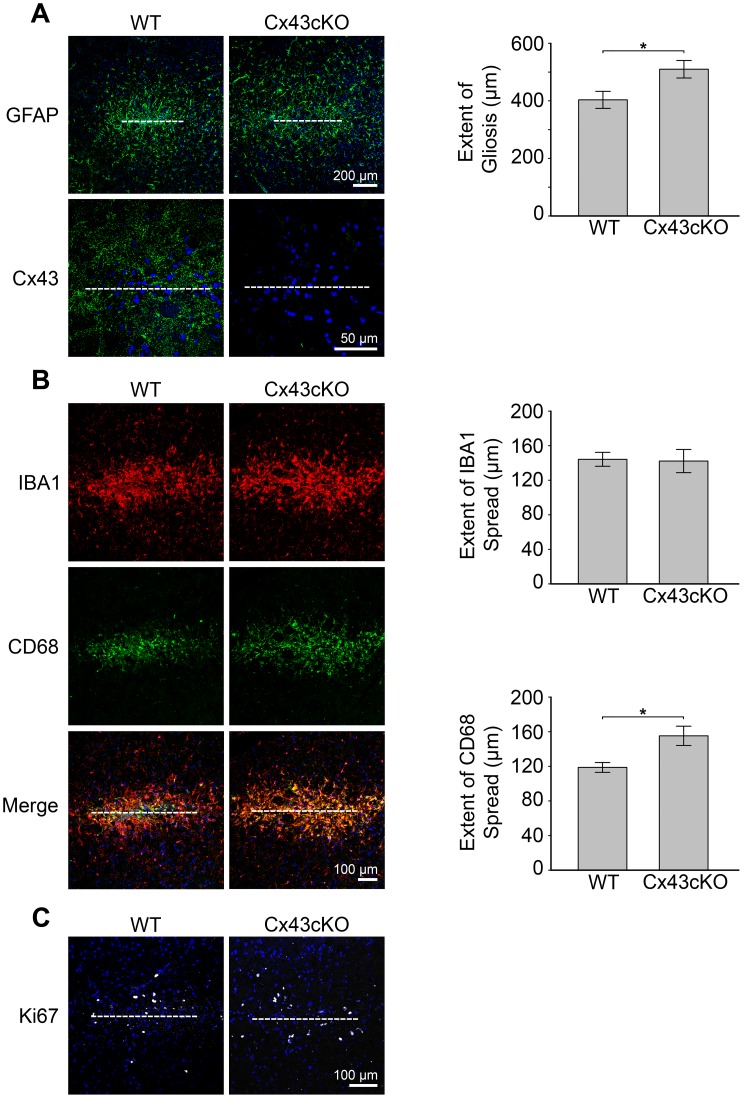
Increased astrogliosis in Cx43-deficient brain. A ) *(left panel)* The distribution of GFAP-expressing reactive astrocytes in response to a needle stab lesion at 6 day post injury (dpi) in Cx43-expressing (WT) and Cx43-deficient (Cx43cKO) brains. Anti-GFAP antibody was used at a concentration to detect only reactive astrocytes with enhanced GFAP expression. *(right panel)* The extent of gliosis at 6 dpi was determined by measuring the width of GFAP immunoreactivity from the needle track. Data were pooled from 3(WT) and 2(Cx43cKO) mice for each time point. * = p<0.05. **B**) *(left panel)* The distribution of IBA1-expressing microglia and CD68-positive cells in response to a needle stab lesion at 6 dpi in WT and Cx43cKO brains. *(right panel)* Graphical representation of the spread of total microglia (IBA1-positive) and reactive microglia/macrophage (CD68-positive) at 6 dpi. The extent of IBA1 or CD68 spread was determined by measuring the width of enhanced IBA1 and CD68 immunoreactivity from the needle track. Data were pooled from 3(WT) and 2(Cx43cKO) mice for each time point. * = p<0.05. **C** *)* Ki67-positive proliferating cells 6 dpi in WT and Cx43cKO brains. Blue, DAPI nuclei staining.

To determine whether the injury response due to stab wound is affected by Cx43 expression, we carried out the same needle injury in GFAP-Cre:Cx43fl/fl mice (Cx43cKO) of which the Cx43 protein was specifically eliminated in neural progenitors and astrocytes [32], [33], [34]. For this series of experiments, we also examined the presence of microglia at an earlier time point of 3 hours post injury (hpi) to decipher whether the absence of Cx43 will affect the initial recruitment of microglia to the wound. In contrast to a previous finding that shows the attenuation of microglial response due to Cx43 inhibition [35], we observed microglial processes extending perpendicular to the edge of the open wound in both wild type (WT) and Cx43cKO brains at 3hpi (Figure 5A). Due to low density of microglia at 3 hpi, we were able to count the number of nuclei associated with IBA1 reactivity along the open wound and detected no difference in the number of IBA1-positive nuclei between them (Figure 5A). Interestingly, there was no GFAP and Ki67 immunoreactivity at 3 hpi in both WT and Cx43cKO brains, indicating the lack of astrogliosis and proliferation within the lesion at such an early time point after injury (Figure 5B). Colocalization of Cx43 immunoreactivity to weak GFAP-positive cells was observed at a region further away from the injury site at 3 hpi (Figure 5C). In addition, some Cx43 was detected as large cytoplasmic aggregates at 3 hpi in IBA1-positive microglia (Figure 5C) with morphology that resembled phagocytic cells [7], suggesting these Cx43 staining may originate from engulfed astrocytes. Taken together, our results suggest microglia activation precedes astrogliosis and agree with a previous observation made in a scalpel wound injury model [1]. At 6 dpi, increased GFAP staining was observed in both WT and Cx43cKO brain (Figure 6A). Interestingly, the extent of astrogliosis was significantly larger in Cx43cKO (509.8±30.4 µm) than in WT (403.4±29.5 µm) brains (Figure 6A). When we assessed the spatial distribution of CD68, which labeled reactive microglia, there was a corresponding increase of CD68 spread in Cx43cKO (155.19±11.16 µm) compared to WT (118.73±5.65 µm) brains (Figure 6B). However, the microglia population, as identified by IBA1 antibody, remained constant (Figure 6B). Finally, there appeared to be no significant difference in the number of proliferating cells between Cx43cKO and WT brains (Figure 6C).

### Cx30 is not Increased at the Needle Lesion

**Figure 7:**
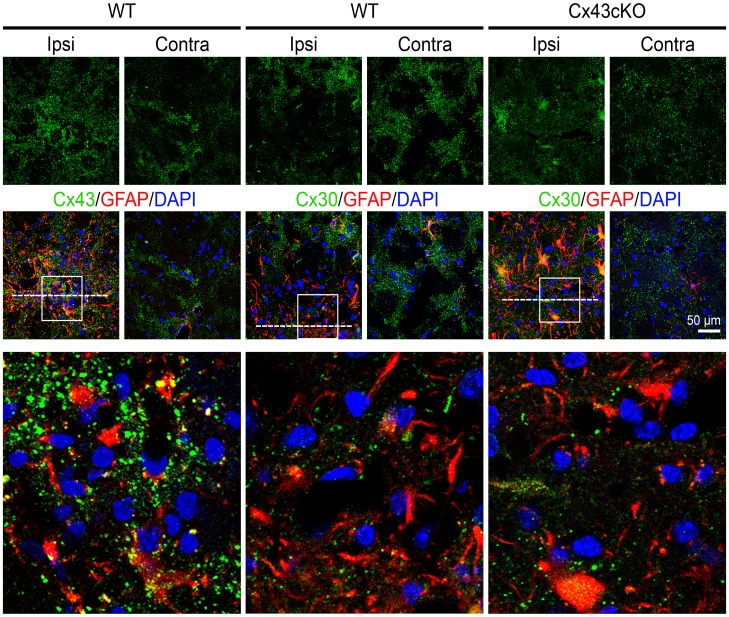
Absence of Cx30 upregulation in response to needle wound injury. Mouse coronal sections were co-stained with anti-Cx30 and anti-GFAP to visualize reactive astrocytes expressing Cx30 at the needle wound 6 days post injury (dpi) in Cx43-expressing (WT) and Cx43-deficient (Cx43cKO) brains. The bottom panel is a magnification of the white boxes in the middle row showing punctate staining of Cx43 and Cx30 at the injury sites. There was no obvious difference in Cx30 immunoreactivity around the wound compared to the corresponding contralateral region. White line denotes position of needle track. Blue, DAPI nuclei staining.

We also examined the kinetics of Cx30, another gap junction protein highly expressed in astrocytes [30], [36], [37], [38]. In contrast to Cx43, no increase in Cx30 immunoreactivity was observed at the peri-lesion area compared to the corresponding contralateral region in both WT and Cx43cKO brains (Figure 7).

### Transient Co-expression of Cx43 with Nestin at the Needle Lesion

**Figure 8:**
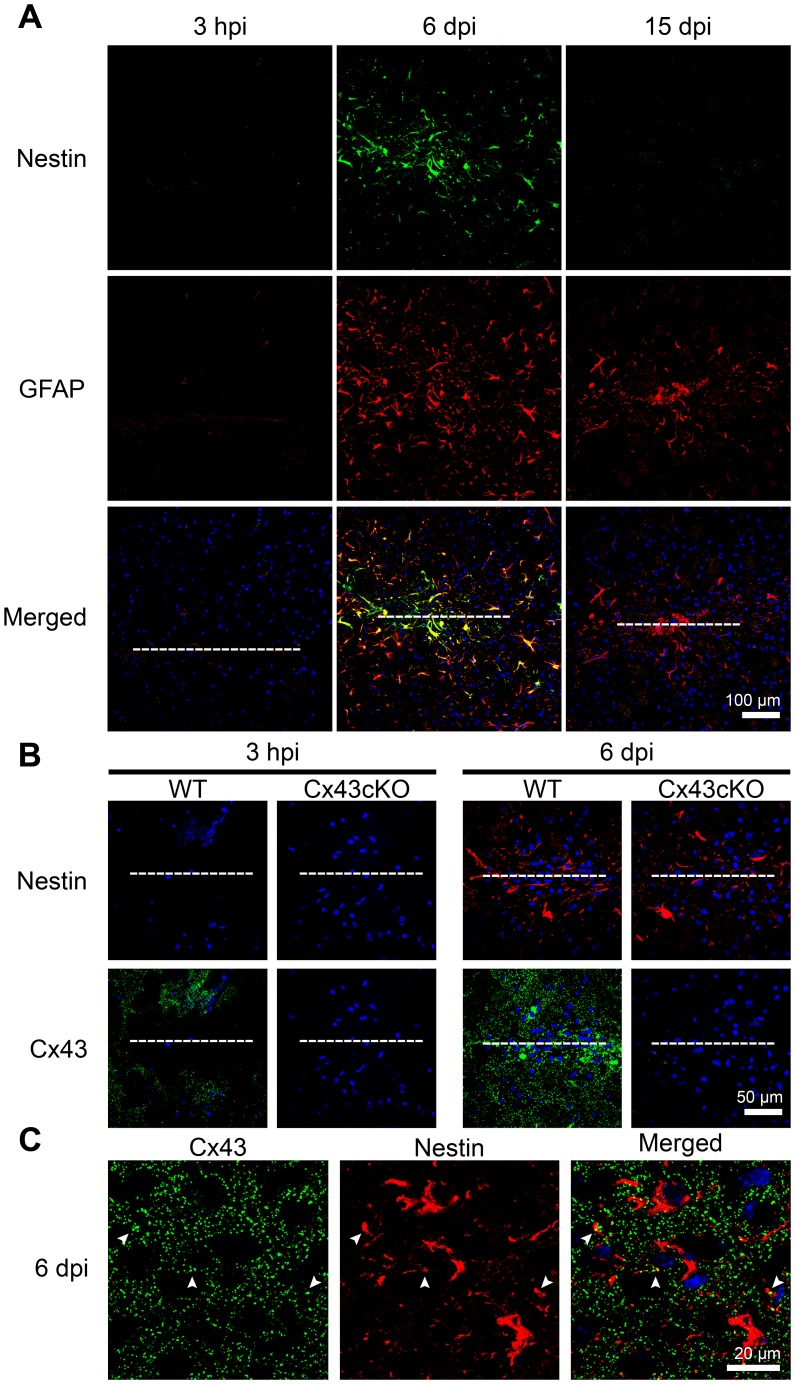
Expression of Cx43 in nestin-expressing cells. **A**) Co-staining of nestin and GFAP markers at the injury site of wild type (WT) brains showed a transient increase in the number of cells expressing both proteins at 6 day post injury (dpi). There was no nestin expression at the wound vicinity at 3 hour post injury (hpi). At 15 dpi, nestin staining was no longer observed while GFAP immunoreactivity was still present at the needle track. Anti-GFAP was used at a concentration to detect only reactive astrocytes with upregulated GFAP expression. White dotted line denotes location of needle wound. **B**) WT and Cx43 knockdown brain of GFAP-Cre:Cx43 fl/fl mice (Cx43cKO) showed similar upregulation of nestin at 6 dpi but no nestin expression at 3 hpi, indicating that nestin dynamics is not dependent on Cx43 expression in astrocytes. Cytoplasmic auto-fluorescence associated with the wound lesion was observed in some sections. **C**) Confocal images showing colocalization of Cx43 puncta with nestin intermediate filaments (arrowheads) in WT brain at 6 dpi. Blue, DAPI nuclei staining.

Nestin neurofilament is expressed in neural progenitor cells and reactive astrocytes following brain insults [39], [40], [41], [42], [43]. In our injury model, we did not detect nestin expression around the lesion sites at 3 hpi but a population of cells positive for both GFAP and nestin appeared at 6 dpi in WT mice (Figure 8A). Interestingly, nestin expression disappeared completely from the injury site at 15 dpi (Figure 8A), which agrees with a recent report using a different injury model [44]. This is in contrast to the sustained GFAP expression detected at 15 dpi (Figure 1, Figure 8A). The spatiotemporal profile of nestin in Cx43cKO brain was identical to the response in WT brain (Fig. 8B). Finally, co-localization of nestin with large Cx43 puncta was observed in the WT brain (Figure 8C), suggesting that Cx43 was expressed in GFAP- and nestin-expressing reactive astrocytes at the needle lesion.

### Cx43 Proteins are not Localized to NG2-glia

**Figure 9:**
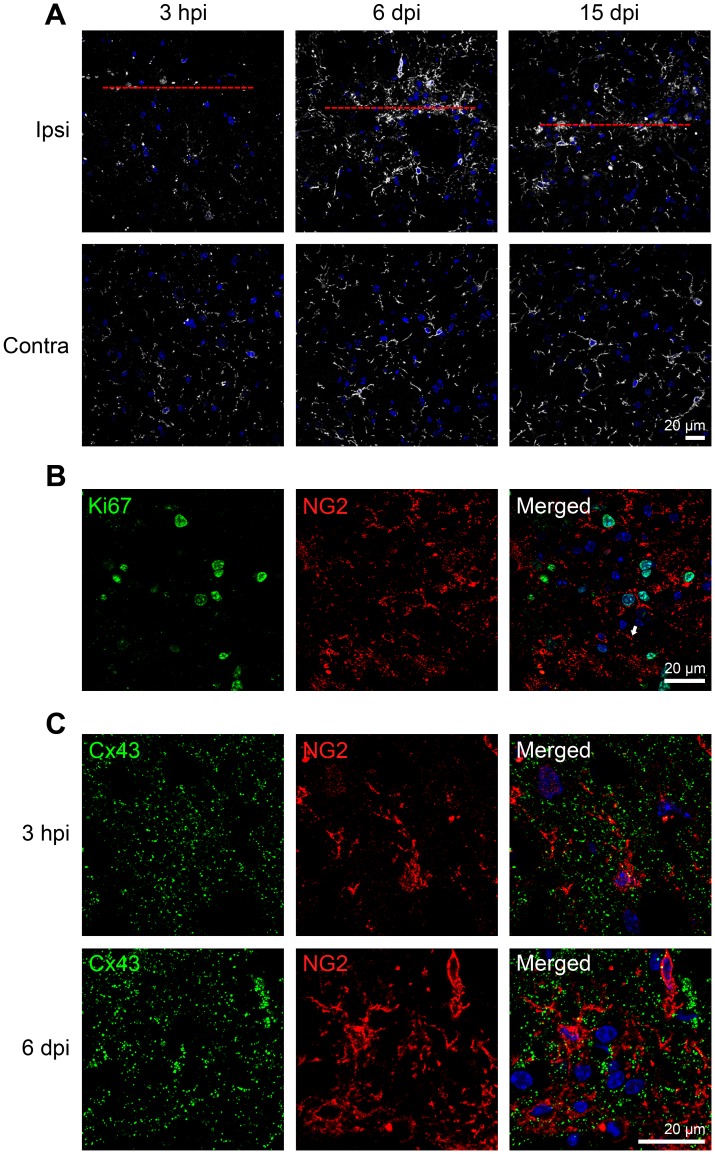
Transient upregulation of NG2-glia in response to needle injury. A ) Immunohistochemical staining of NG2-glia or oligodendrocyte precursor cells by NG2 antibody. Increased NG2 immunoreactivity at and around the lesion was observed at 6 day post injury (dpi) in wild type mice. There was no increased NG2 expression at 3 hour post injury (hpi). NG2-glia was back to near basal level (when compared to contra-lateral hemisphere) by 15 dpi. Cytoplasmic auto-fluorescence was observed in the needle track at 15 dpi. Red line denotes position of needle track. **B**) Co-staining of Ki67 proliferative marker with NG2 antibody at 3 dpi, indicating some NG2 cells were proliferating. **C)** Co-staining of Cx43 with surface proteoglycan NG2 showed minimal localization of Cx43 with NG2-positive cells. Blue, DAPI nuclei staining.

Some reports have indicated that astrocytes can be generated from OPCs or NG2-glia after injury [2], [3]. Therefore, we looked into the possibility that Cx43 may be expressed by NG2-glia in WT mice. Similar to the GFAP or nestin expression profile, an increase in NG2 staining was not apparent at 3 hpi when compared to the contralateral side but became evident at 6 dpi (Figure 9A). Like nestin, NG2 reactivity appeared to return to basal levels at 15 dpi, which is in agreement with a previous report [4] although a separate study showed elevated accumulation of NG2-positive cells around the lesion site 14 days after stab wound injury [1]. This discrepancy may be explained by the difference in the severity of inflicted injury. We also detected proliferating NG2-glia that were Ki67 positive at 6 dpi (Figure 9B), consistent with previous reports [1], [4]. Double staining with anti-Cx43 and anti-NG2 antibodies did not reveal extensive colocalization at 3hpi nor 6dpi (Figure 9C). Taken together, our results suggest the increased immunoreactivity of Cx43 at the lesion site was contributed primarily by astrocytes and not microglia or NG2-glia.

## Discussion

Astrogliosis is observed around brain lesions due to ischemia and mechanical injuries such as a stab wound [8], [9], [10], [11]. It is characterized by increased proliferation of reactive astrocytes and a concomitant increase in secretion of growth factors [11], [45]. Hypertrophy of astrocytes is always accompanied by an upregulation of GFAP intermediate filament [8], [9]. Although the functional significance of enhanced GFAP expression is unclear, mice that are deficient in both GFAP and vimentin exhibit reduced gliosis [46], [47], [48]. Similarly, there is an increased detection of Cx43 in astrogliosis and selective elimination of Cx43 in astrocytes causes a reduction of gliosis in brain ischemia and spinal cord injury models [49], [50], [51]. In spite of its close association with reactive astrocytes, Cx43 has not been widely used as an indicator for astrogliosis, and even less is known about its role in the formation of reactive astrocytes. In this report, we used a simple stab wound to establish that Cx43 is expressed mainly by GFAP-positive astrocytes and its spatiotemporal profile mirrors the extent of astrogliosis, as determined by GFAP immunoreactivity. The lack of colocalization of Cx43 with IBA1 and NG2 that are upregulated in response to injuries [4], [52] confirms the tight association of Cx43 with astrocytes and not with microglia or NG2-glia. Interestingly, Cx43 also co-localized with nestin-expressing cells, which may be due to re-expression of nestin in a sub-population of de-differentiating astrocytes in response to injury [53]. The presence of immature cells due to proliferation around a needle wound may also explain the lack of Cx30 expression in the area since Cx30 is only expressed in mature astrocytes [54].

There are a few possibilities that account for increased Cx43 immunoreactivity around the injury site. First, the protein can be upregulated by the reactive astrocytes. Second, the increased number of astrocytes around the lesion may result in a cumulative increase in Cx43 immunoreactivity; and third, the antigenicity and subcellular localization of Cx43 may be altered by the injury, as suggested by previous studies [27], [55], [56], [57]. While a report has confirmed the upregulation of Cx43 mRNA surrounding the lesion site [28], a recent study using a genomic approach did not report a significant increase in Cx43 in reactive astrocytes [44]. Although our study cannot distinguish between these possibilities, the fact remains that increased Cx43 immunoreactivity was sustained for at least 2 weeks after injury. The reduction of Cx43 immunoreactivity at 3 dpi at the injury site is an interesting observation; it may indicate the downregulation of the protein by cytokines secreted by microglia, as has been previously reported [58], [59], [60], [61], [62].

Our findings from Cx43cKO brains *in vivo* clearly suggest that astrocytic Cx43 is not required for microglial activation or astrogliosis induced by a needle wound. In fact, our results show that both processes are more extensive in the brains lacking Cx43. The upregulation of Cx43 protein in the lesion site about 1 week after injury agrees with a role of Cx43 in ‘wound healing’ to facilitate the integration of newly-formed cells [15], [58], [63] instead of participating in the initial inflammatory responses. Although Cx43 has been detected in activated microglia [64], [65], [66], [67], others have also demonstrated the lack of Cx43 expression in microglia [28], [59], [68]. It is likely that phagocytosed astrocytic Cx43 account for large cytoplasmic aggregates observed within the microglia at 3hpi. Alternatively, Cx43 expression is proposed to correlate with extent of microglial activation [69] and it remains a possibility that the needle injury is not sufficient to trigger upregulation of Cx43 in microglia. Interestingly, a recent study demonstrates the weakening of blood-brain barrier (BBB) due to deletion of Cx43 and Cx30 [70]. Therefore, it is possible that enhanced CD68 immunoreactivity in the Cx43cKO brain may indicate the increased number of macrophages [71] due to breaching of the BBB, which subsequently leads to more severe astrogliosis. Our findings of increased astrogliosis contrasted with other studies [15], [49], [51]. We speculate the relatively minor and localized injury of our model when compared with global damage in other injury models may account for the discrepancy.

Taken together, our study confirms that Cx43 is specifically associated with reactive astrocytes. We also demonstrate that an injection or needle biopsy in the brain is sufficient to induce inflammatory responses similar to those activated by severe injuries. Although Cx43 expression generally mirrors GFAP immunoreactivity spatially and temporally, there is one important difference. GFAP expression remained upregulated in the peripheral region when compared to the contralateral hemisphere for the entire 15-day period post injury. In contrast, upregulation of Cx43 level at the peripheral region quickly returned back to basal level and remained high only specifically at the lesion site, indicating Cx43 will be a better marker to visualize a ‘recovering’ wound. GFAP has been used as biomarker for traumatic brain injury [72]. The other commonly used markers for reactive astrocytes include other intermediate filaments such as nestin and vimentin [11]. Some recent markers for astrogliosis include S100β, Musashi, Synemin and Bystin [73], [74], [75], [76] which are either cytoplasmic or nuclear proteins. As a membrane protein, Cx43 has an additional advantage due to its accessibility for antibody or peptide binding. An example of a potential *in vivo* clinical application has been demonstrated in rodents using an antibody that recognizes the extracellular loop of Cx43 [77]. Unlike the extensive injuries inflicted by earlier studies [1], [78], the small injury in our model reflects early stages of brain diseases. Therefore, the possibility to detect increased Cx43 reactivity specifically in a small lesion will be useful for visualizing asymptomatic brain damage.

## Materials and Methods

### Ethics Statement on Animal Experiments

All breeding and animal procedures were approved by The University of British Columbia Animal Care Committee (Protocol No: A10-0266) and performed in accordance with the guidelines established by the Canadian Council on Animal Care.

### Animals

Cx43 conditional knockout mice were generated by crossing GFAP-Cre mice [32] with mice harboring floxed Cx43 alleles [33]. We then identified conditional knockouts by PCR genotype analysis as described in detail previously [79]. The mice were maintained in an animal facility for 12 hr light/dark cycle, and were provided food and water *ad libitum*.

### Intracerebral Needle Injury

Adult C57BL/6 mice aged three months were anaesthetized and placed on a stereotactic head holder. A hole of 1.0 mm diameter was drilled through the skull and 3 µl phosphate buffered saline (PBS) mixed with Indian ink (to visualize the lesion site) was injected intracerebrally with a 33 gauge syringe into the striatum at the position of 2.5 mm lateral to the midline, 1.0 mm anterior of the bregma, and 3.0 mm ventral from the dura. The hole in the skull was resealed with wax and mice were allowed to recover. At 3, 6, 9, and 15 days post injury, brains were pre-fixed by transcardial perfusion with 4% paraformaldehyde in 0.1 M PBS prior to removal, and subsequently post-fixed after removal by immersion in the same fixative for 8 h at 4°C. Fixed brains were then equilibrated with phosphate-buffered 30% sucrose, mounted in OCT (Tissue-Tek) and cut into 10 µm coronal sections using a cryostat. To examine the initial host response to the needle injury, we similarly processed brains at 3 hours post injury.

### Immunofluorescence

Cryosections were probed with the following primary antibodies: mouse IgM anti-connexin43 (1∶400; Sigma); rabbit anti-connexin43 (1∶800; Sigma); rabbit anti-connexin30 (1∶100; Invitrogen); rabbit anti-IBA1 (1∶400; Wako); rat anti-CD68 (1∶200; Serotec); mouse anti-GFAP (1∶800; Sigma); rabbit anti-GFAP (1∶200; Sigma); mouse anti-Ki67 (1∶200; BD Pharminogen); rabbit anti-NG2 (1∶100; Millipore), and mouse anti-Nestin (1∶100; Developmental Studies Hybridoma Bank). Cryosections were briefly washed with PBS for 5 min and blocked for 1 h in 2% BSA and 0.3% Triton X-100 in PBS at room temperature. For antibodies requiring antigen retrieval (e.g. anti-Ki67), cryosections were subjected to a 10 min incubation in 5 mM citrate buffer at 95°C followed by ice bath cooling for 20 min prior to the blocking step. Sections were then incubated overnight at 4°C with primary antibodies diluted with 1% BSA and 0.3% Triton X-100 in PBS, rinsed twice in PBS for 15 min each and incubated in the appropriate Alexa-Fluor® fluorescent-conjugated secondary antibodies (Invitrogen) for 1 h at room temperature. After rinsing the sections twice in PBS for 15 min, they were mounted with Prolong Gold antifade reagent with 4′,6′-diamidino-2-phenylindole (Invitrogen).

### Image Acquisition and Statistical Analysis

Images were acquired with an Olympus Fluoview FV1000 confocal microscope with a resolution of 640×640 pixels. Laser power, gains and rate of acquisition were optimized for each antibody and remained constant for the entire series of 3, 6, 9, and 15 dpi. Quantification of GFAP, IBA1 and Cx43 immunostaining was achieved by manually thresholding the captured images with Image J software [80] so that puncta with radiance above a certain value (i.e. threshold) were identified. This threshold value was subsequently used to quantify all images for the entire time series from the same antibody. Size of the marked particles must be above 3 µm^2^ and 5 µm^2^ for GFAP and IBA1 staining respectively to be included in the final analysis. Cx43 had small puncta staining and all puncta were therefore included. Measurements were sampled separately from three non-overlapping areas of 20000 µm^2^ each: a central region that includes the needle lesion (100 µm from the needle track), a peripheral zone adjacent to the central region and the corresponding contralateral region (see Figure 3B). The final value was presented as fluorescence density in mean ± SEM. The level of proliferation was presented as the density of Ki67-positive nuclei associated with GFAP (astrocytes) or IBA1 (microglia) staining in an ellipse with a major axis of 300 µm plus the length of needle track, and a minor axis of 300 µm. Extent of gliosis and microglia spreads (as visualized by immunostaining of GFAP, IBA1 and CD68 respectively) were measured using Axiovision software with images acquired by a Zeiss Axioskop epifluorescence microscope. Number of microglia at the stab wound lesion was quantified by counting the number of IBA1-positive nuclei lining the wound per 250 µm length. Statistical analysis was carried out with student’s 2 tailed *t* test or Mann-Whitney U-test.
